# 188. Plan PDD (patient directed discharge): Oral contingency plans for people who inject drugs (PWID) with *Staphylococcus aureus* bacteremia (SAB) who leave the hospital prematurely

**DOI:** 10.1093/ofid/ofad500.261

**Published:** 2023-11-27

**Authors:** Olivia Duffield, Kaya Patel, Sara K Schultz, Stephanie Spivack

**Affiliations:** Temple University Hospital, Philadelphia, Pennsylvania; Temple University Hospital, Philadelphia, Pennsylvania; Temple University Hospital, Philadelphia, Pennsylvania; Temple University Health System, Philadelphia, Pennsylvania

## Abstract

**Background:**

There has been an increase in infectious complications in PWID and SAB continues to be a serious consequence with treatment options that may not align with patient's agency. High rates of discharges against medical advice and subsequent readmissions contribute to preventable morbidity and mortality plaguing this stigmatized population. Recent research has indicated that oral antibiotic therapy can be non-inferior to IV therapy for SAB infections.

**Methods:**

We queried Epic for admissions with patients with documented opioid use disorder and injection behavior and positive blood cultures from September of 2021 to March of 2022 at our large academic medical center in Philadelphia. We conducted a retrospective chart review of these encounters and collected data regarding their hospital course and treatment.

**Results:**

Over the six-month study period, there were 159 admissions for bacteremia in PWID. Over half (52%) of these patients left the hospital prior to completing IV treatment, 24 (15%) of which still had positive blood cultures at the time of discharge. 90 patients had SAB infection (56.6%). In patients with SAB, only 23 (25.5%) completed a course of IV antibiotics while 52 (57.8%) patients with SAB left the hospital in a PDD and did not complete a course. In addition, there were 6 in-hospital mortalities (6.7%). Of the 52 patients that left the hospital without completing IV antibiotics, 21 (40.4%) were offered an oral antibiotic alternative. For patients who received an oral antibiotic on discharge, only 2 (9.5%) patients were readmitted in 90-days compared to 11 (35.5%) readmissions if oral antibiotics were not received (Figure 1). 19 patients (22.6%) total with SAB were readmitted within 90 days for the same infection or a complication of inadequately treated bacteremia.

Figure 1

**Figure:**
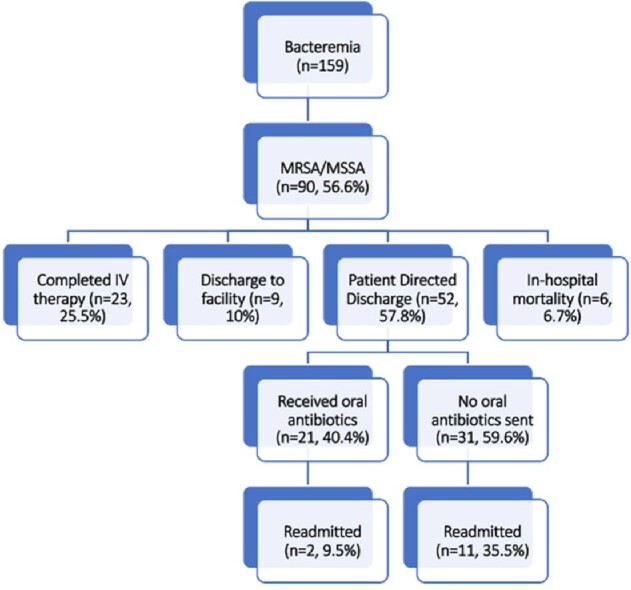

Outcomes for PWID with SAB

**Conclusion:**

PWID with SAB experience significant challenges to remaining in a hospital setting for the prescribed duration of intravenous antibiotics. ID physicians should consider including a contingency plan for oral antibiotics should the patient leave the hospital prematurely. Our results show that oral antibiotics are a viable option to prevent 90-day readmission in PWID with SAB.

**Disclosures:**

**Sara K. Schultz, MD FACP FIDSA**, AbbVie: Advisor/Consultant

